# Additive Manufacturing of Micro-Electro-Mechanical Systems (MEMS)

**DOI:** 10.3390/mi12111374

**Published:** 2021-11-08

**Authors:** Giorgio De Pasquale

**Affiliations:** Department of Mechanical and Aerospace Engineering, Smart Structures and Systems Lab, Politecnico di Torino, Corso Duca degli Abruzzi 24, 10129 Torino, Italy; giorgio.depasquale@polito.it

**Keywords:** additive manufacturing, 3D printing, MEMS, microstructures, digital manufacturing, IOT

## Abstract

Recently, additive manufacturing (AM) processes applied to the micrometer range are subjected to intense development motivated by the influence of the consolidated methods for the macroscale and by the attraction for digital design and freeform fabrication. The integration of AM with the other steps of conventional micro-electro-mechanical systems (MEMS) fabrication processes is still in progress and, furthermore, the development of dedicated design methods for this field is under development. The large variety of AM processes and materials is leading to an abundance of documentation about process attempts, setup details, and case studies. However, the fast and multi-technological development of AM methods for microstructures will require organized analysis of the specific and comparative advantages, constraints, and limitations of the processes. The goal of this paper is to provide an up-to-date overall view on the AM processes at the microscale and also to organize and disambiguate the related performances, capabilities, and resolutions.

## 1. Introduction

Additive manufacturing (AM) and 3D printing are consolidated processes for the production of components at the macroscale at the industrial level, although many optimization issues still remain open and motivate academic research. Instead, the AM of microstructures and MEMS (micro-electro-mechanical systems) is based on dedicated processes that are still under development and validation. In particular, some relevant process performances such as accuracy, resolution, and repeatability are not fully consolidated, and materials availability is still limited [1,2,3,4,5,6,7,8,9,10]. The AM processes are classified into seven categories according to the international standard [11]: binder jetting, directed energy deposition, material extrusion, material jetting, powder bed fusion, sheet lamination, and vat photopolymerization. By starting from the original processes classification, many variations were introduced and original methods were developed. As a result, a very high number of fabrication methods are available today, with different levels of maturity and reliability. Similarly, many associated acronyms can be found, sometimes with the same meaning.

The development of a MEMS based on 3D printing and AM always needs a specific design approach. The knowledge of the available process typologies, combinations, performances, and available materials is mandatory to improve the manufacturability and sustainability of micro 3D-printed devices. The common advantage of all AM methods is the direct building from the digital geometry file or model (computer-aided design, CAD) to the real component. This conversion is possible with wider shape freedom than with conventional micromachining building processes [7,12]. At the present level of development, some AM methods demonstrated resolutions up to the nanometer range and improved the quality of structures in terms of surface finishing and parts geometry. The integration of AM methods into high-performance technologies (nanoimprint lithography, roll-to-roll processing, etc.) is possible, as the development of combinations of different additive methods.

The AM available materials are increasing in number, increasing those ones with functional properties. The compatible materials are polymers (polyamide, acrylates, polylactic acid or “PLA”, acrylonitrile butadiene styrene or “ABS”, epoxy resins, polycarbonate) [13], metals (titanium, aluminum and nickel alloys, stainless steel) [14], ceramics (alumina, lead zirconate titanate or “PZT”, silicon carbide, titanium dioxide) [15], and soft materials (hydrogel, liquid crystals, polydimethylsiloxane or “PDMS”) [16]. Many AM methods show recurring issues related to dimensional accuracy and thermal shrinkage, especially in polymer-based processes, ceramic green parts sintering, and metal parts fabrication.

The emerging AM technologies applied to the micro- and nanoscales [3,6] demonstrate preliminary applications in mechanics [17], electronics (sensors, light-emitting diodes-LEDs) [18,19], optics and photonics (filters, photonic crystals, meta-materials, diffractive elements) [20,21,22], medicine [23] and bionics [24,25,26]. The rise of AM is expected in some emerging fields of microstructures such as wearable electronics [27,28], flexible batteries management, internet of things (IOT) [29], printed bionics/biomechanics [19], lab-on-chip [30], and self-powered sensors.

## 2. Classification of AM Processes for MEMS

The comprehensive review of the presently available AM processes suitable for the microscale can be divided into four categories: powder-based processes, other laser-based processes, extrusion-based processes, and other processes. The next sessions report the description of these processes, the associated materials, and features size. The differences among the process variants are reported and the multitude of acronyms available in the literature are disambiguated.

The nomenclature of AM processes is subjected to frequent revisions and updates, under the guidelines given by ASTM standards. However, many acronyms used in the past, although formally outdated, are still commonly used in scientific papers and technical reports due to their clear technological significance. For example, the *selective laser melting (SLM)* process has been re-named as *laser-based powder bed fusion of metals (PBF-LB/M)* and *laser-based powder bed fusion of polymers (PBF-LB/P)* [31]. In this paper, the AM processes nomenclature cited is not limited to the international standards presently active and also includes widely used terms and definitions.

**Figure 1:**
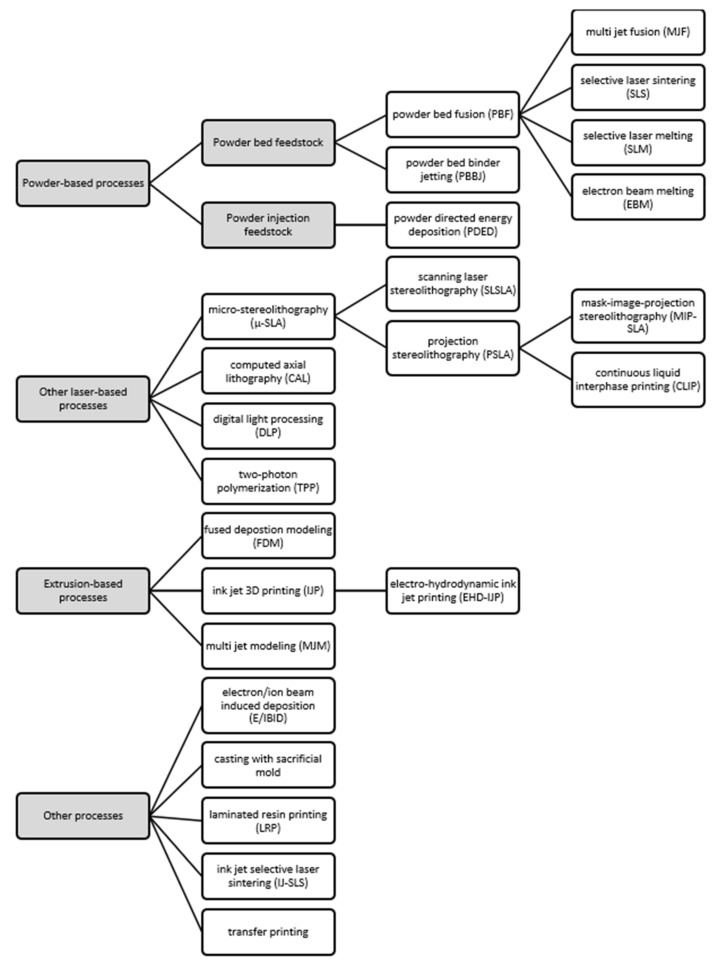
Classification of the AM processes for MEMS fabrication.

The powder-based AM processes refer to the presence of powder bed or powder injection feedstock. The laser-based processes (excluding those already described in the first category) refer to laser power sources. The extrusion-based processes identify the building growth through extruded layers of materials. Finally, the other processes not included in the previous categories are reported. The block diagram of Figure 1 reports the AM processes for the microscale organized in their respective categories.

## 3. Powder-Based Processes

The AM processes based on powders management are suitable to many compatible materials, provide fast production speed thanks to the limited supports needed, and offer high accuracy. For these reasons, they have a high potential for MEMS fabrication. Another advantage is the reduced waste materials due to the reusability of unprocessed powder [4]. With reference to the seven AM methods mentioned in the standards [11], the powder-based processes include three of them: *powder bed fusion (PBF), powder directed energy deposition (PDED),* and *powder bed binder jetting (PBBJ)*. Other variants of these processes have been developed: the *multi jet fusion (MJF),* the *selective laser sintering (SLS),* the *selective laser melting (SLM),* and the *electron beam melting (EBM)*. In all cases, the powder is processed by starting from a powder bed feedstock (metals and polymers) or from a powder injection, or blown powder, feedstock (metals only).

The challenges and limitations of this group of processes in the MEMS field are associated with the resolution of the energetic source and the powder granulometry. The average spot size of laser beams or electron beams is able to produce structures with dimensions largely below 500 μm, generally down to 100 μm. The surface roughness is critical because for MEMS the post-process mechanical tooling is generally not applicable, and the “as-built” accuracy is the only one available. For MEMS, the process parameters are much more sensitive than in the macro-scale, in terms of laser speed, layer thickness, and laser power (contributing to the definition of the volume energy density, VED). The combination of these parameters affects the so-called “up-skin” and “down-skin” printing resolution, referred to as the horizontal planes. Furthermore, in the microscale, the printing parameters variation between the contour (the perimeter of the structure) and the inner parts is very critical due to the small structure dimensions. Here, the fast and high gradient of the VED may produce local melting/sintering issues (distortion and porosity).

### 3.1. Powder Bed Fusion (PBF)

The *powder bed fusion (PBF)* method was introduced the first time by Deckard and Beaman [32]. It is based on a feedstock where the powder is exposed selectively to a descendent energy source that causes the powder to sinter or melt [33,34]. Depending on the energy source (laser, electron beam, infrared, thermal) and powder pre-heating, the process gets different names. The materials compatible with PBF processes are metals, polymers, and ceramics, although other typologies can be used as composite powders, calcium carbonate, and sand. With exposure to the energetic source, the powder is locally sintered or melted and the object is created layer by layer. Vertically movable stages and powder re-coaters are needed to run the process into confined chambers with a controlled atmosphere. When the structure is completed, the un-exposed powder is removed and possibly reused. The standard manufacturing guidelines indicate a minimum feature size of 500 μm for PBF of polymers and metals [35]. The next sessions (from 3.2 to 3.5) describe the variants of the PBF process.

### 3.2. Multi Jet Fusion (MJF)

**Figure 2:**
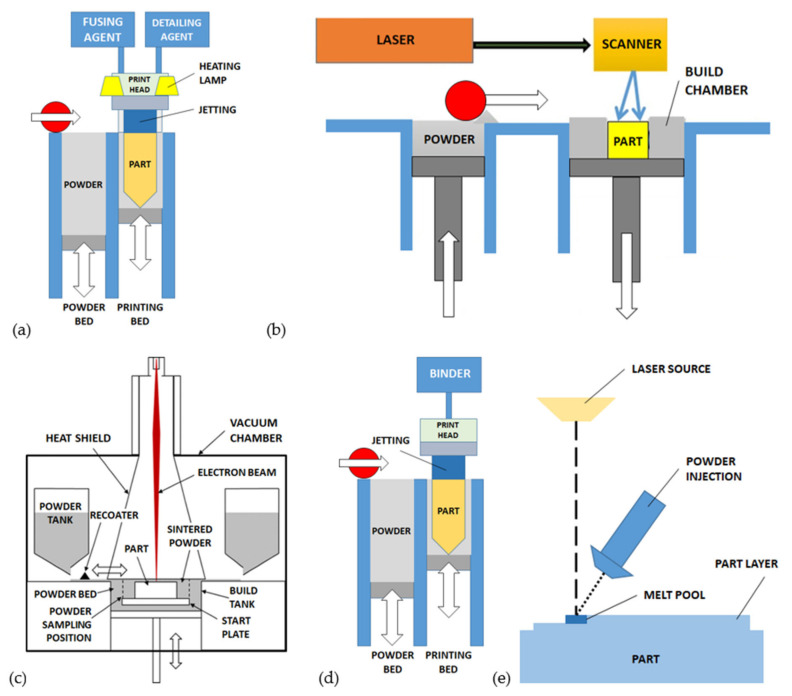
Powder-based processes for AM of microstructures: (**a**) multi jet fusion (MJF), (**b**) selective laser sintering/melting (SLS/SLM), (**c**) electron beam melting (EBM), (**d**) powder bed binder jetting (PBBJ), (**e**) powder directed energy deposition (PDED).

The *multi jet fusion (MJF)* process, introduced by Hewlett-Packard (HP), belongs to the PBF category and operates with polymers [36,37,38]. Generally, it is used for low volumes and fast components production with high mechanical strength as an AM alternative to the traditional injection molding [34,36,39,40]. The powder feedstock is similar to the other PBF processes but the polymer material is fused by using an infrared heating source combined with chemical agents. The print surface is pre-heated to a uniform temperature and then a thin layer of powder is deposited on it (Figure 2a). The HP thermal inkjet of the printing head is then used to deposit on the powder layer a combination of fusing and detailing agents on different selected areas. After that, the print surface is exposed to an infrared source and the powder is fused only where the fusing solution (i.e., radiation absorbing agent) is present [34,36,41,42]. The available polymers for MJF are polyamide (PA 11, PA 12) and thermoplastic polyurethane (TPU). MJF is suitable for printing functional mechanical parts or devices, biomedical lattices structures, medical orthotics and prosthetics, mechanical tools, and fluid-tight devices [36,39,43,44,45,46,47,48].

### 3.3. Selective Laser Sintering (SLS)

In this process, a laser beam is directed against the upper surface of the powder as represented in Figure 2b. The SLS is the most common AM process for polymers (nylon, polycarbonate, polymer composites, etc.), although metals, ceramics, hydroxyapatite, and glasses are also compatible [34,49,50,51]. The laser-scanning path is obtained from the processing and slicing of the digital 3D drawing. The 3-dimensional object is obtained as the result of layer-by-layer fusion and sintering of the powder, by eventually pre-heating the material and by using inert atmosphere into the printing chamber. The vertical stage is moved downward, a powder layer is deposited by the recoater arm and then exposed to the laser source [52]. The maximum accuracy of the resulting SLS features is in the range 40–100 μm [10,53,54,55].

The definition of SLS is associated with a large variety of materials, including metals. However, in this case, the alternative names of *direct metal laser sintering (DMLS)* or *direct selective laser sintering (DSLS)* are preferably used in literature and practice [52,56]. The documented typical feature size of DMLS is around 500 μm, although the minimum documented values reached 380 μm and 153 μm for standard and high-resolution processes [57].

### 3.4. Selective Laser Melting (SLM)

The SLM process is addressed to metals, and it is also referenced as *direct metal laser melting (DMLM)* or *laser powder bed fusion (LPBF)*. It is very similar to SLS, but the powder particles are heated until the full melting by using laser beams with higher power (Figure 2b) [58,59,60,61,62]. This difference provides higher mechanical strength of the processed materials than SLS [63,64,65]. The process sequence is similar to SLS, where the layer thickness ranges between 20 and 100 μm and the metal powder granulometry is about 20–50 μm [66]. The minimum feature size reported for SLM is 40–200 μm [67,68]. Some examples of micro-channels fabricating with SLM in steel parts with 120 μm wall thickness and square cross-section were documented [69]. In glass material, SLM was used to build channels with 1.1 mm cross-section [70]. The so-called micro-SLM process, recently developed achieved microscale features of 60 μm and 1.3 μm minimum surface roughness (Ra) [66].

### 3.5. Electron Beam Melting (EBM)

The EBM process is applied exclusively to pre-heated metal powders in a vacuum chamber. As reported in Figure 2c, an electron beam heat source is used to fully melt the powder grains [71]. The pre-heating increases the stability and compactness of powder, then suspended parts can be obtained with reduced supports density, as a general rule. The process resolution is in the range 100–200 μm [72,73]. As an example, different versions of scaffolds with gyroid shape and composed of unit cells with a minimum feature size of 500 μm were built [74].

### 3.6. Powder Bed Binder Jetting (PBBJ)

The PBBJ is applicable to a large variety of materials, including polymers, metals, sand, ceramics, and mixtures of them [75,76,77,78,79,80]. The powder grains are deposited together with a liquid binding agent working as cohesive media (Figure 2d). The layers are sequentially deposited upon a platform that moves progressively downwards. The result of the first phase of the process is a 3D object called the “green part”. The green part is then subjected to extensive post-processing, including debinding, sintering and, eventually, infiltration and hipping (hot isostatic pressure) to remove the binder solution and increase the mechanical strength by reducing porosity [76,77,79,81,82,83,84,85]. In the case of ceramics, the resolution of PBBJ is largely variable in the range 22–500 μm [86,87], while in the case of metals and polymers the typical feature size is about 100 μm [13], with variations related to the powder grains dimension [86]. In fact, the performances of surface roughness and mechanical resistance increase with finer powder below 20 μm. The presence of the binder is responsible for accuracy issues in terms of shrinkage and deviations from the nominal dimensions.

### 3.7. Powder Directed Energy Deposition (PDED)

The PDED process is based on injection feedstock of powder supported by robotic systems with multiple axes. The heat source is generally represented by a laser source but other sources can be used (electron beam, plasma, electric arc). The PDED process is also called *direct laser metal deposition (DLMD)*.

The injector and heat source are coupled, then the metal powder injected is immediately melted and deposited on the target surface, where the temperature rapidly decreases and causes the solidification (Figure 2e). The physical and chemical bonding between the target and the deposited materials is obtained [88,89]. The applicable strategies of injection include the lateral and off-axis orientation, and the continuous or discontinuous powder injection [59]. The different injection methods associated with the specific heat source and kinematic system lead to the sub-categories of PDED known as *laser engineered net shaping (LENS), laser metal deposition (LMD), direct laser deposition (DLD), direct light fabrication (DLF), laser deposition welding (LDW)* and *powder fusion welding (PFW)* [88,89].

The mechanical characteristics of the process do not consent to reach high resolutions, which are limited to values included between 500 and 3000 μm [57]. The process variant associated with the lowest values of this range is also defined *micro-PDED/DLMD process (μ-PDED/DLMD)* [90,91,92]. Some features represented by 20 μm single pattern tracks were fabricated by μ-PDED/DLMD [90].

## 4. Other Laser-Based Processes

This category includes all the laser-based processes for MEMS fabrication which are not mentioned in the previous section. In particular, the group of processes based on the photopolymerization of liquid resins are reported. The main challenges of these AM processes are associated with the limitation of resolution due to the optical properties of the polymers used. The light refraction and diffraction aredirectly responsible for the thickness of the transition region between exposed and unexposed liquid volumes. In the MEMS scale, the small liquid volume involved limits the light deviation, then dimensions below 50 μm are easily achievable with high precision. The situation is different for the *two-photon polymerization (TPP)* process, where the pulse light generated by a laser transparent photopolymer is able to expose the resin exactly at the beam focal point, with a precision in the order of a few nanometers.

### 4.1. Micro-Stereolithography (μ-SLA)

The so-called “stereolithography apparatus” (SLA) is the name assigned by Charles W. Hull to the system he patented in 1984 [93], together with the successful STL file format for the digital slicing of 3D models. They became the tool to support the most diffused, cheap, and fast 3D printing method for polymers for many years [6]. The basic principle is to excite with light source a bath of liquid photosensitive material to induce the polymerization and solidification (photocuring) in selected regions [93,94,95,96,97,98].

**Figure 3:**
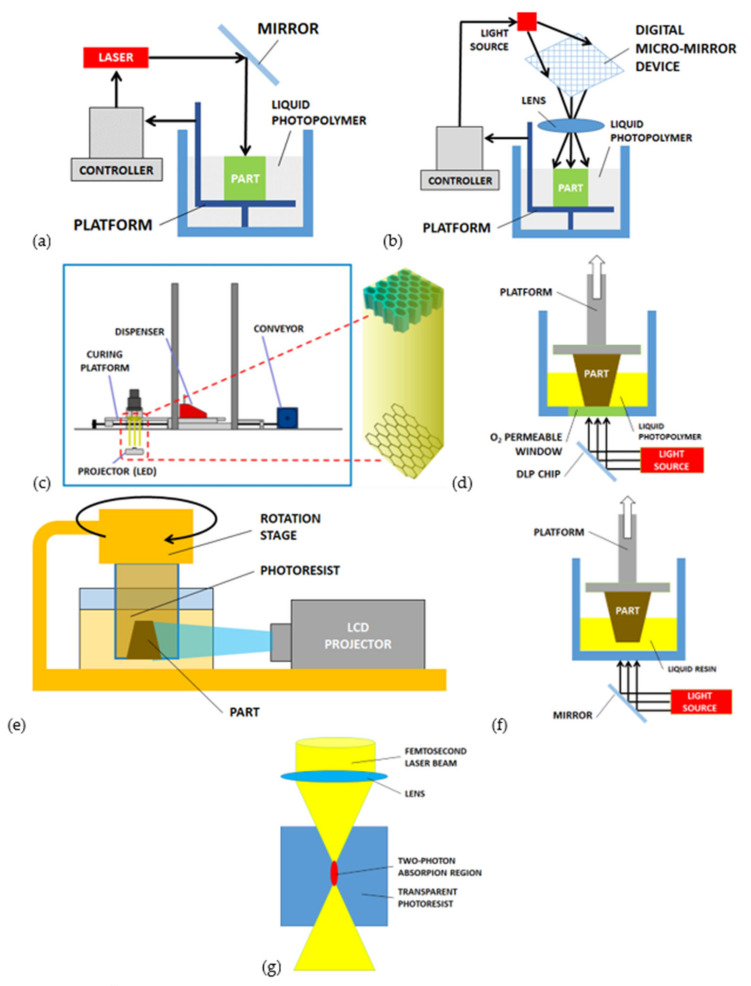
Other laser-based processes for AM of microstructures: (**a**) scanning and (**b**) projection micro-stereolithography (μ-SLA), (**c**) mask-image-projection stereolithography with 3D mask (MIP-SLA) [99], (**d**) continuous liquid interphase printing (CLIP), (**e**) computed axial lithography (CAL), (**f**) digital light processing (DLP), (**g**) two-photon polymerization (TPP) or direct laser writing (DLW).

The two main sub-categories of SLA are defined in relation to the exposure strategy: scanning and light projection [94]. In the *scanning laser stereolithography (SLSLA)*, as reported in Figure 3a, the laser beam produces the local polymerization of the liquid resin point-by-point on its surface. The laser pattern is defined by the digital processing of the original geometry, which is preliminarily sliced to identify the exposure areas corresponding to each layer. The thickness of each layer is in the range 10–100 μm. The layers’ superposition is achievable thanks to a building platform that moves downwards [6]. In the *projection stereolithography (PSLA)* process (Figure 3b), the entire surface of the liquid photosensitive resin is exposed to the light source simultaneously. Digital micro-mirror devices (DMD) composed of a matrix of reflecting and orientable surfaces, individually controllable, provide the shaping of light. The DMD chip may contain several million micro-mirrors, combined together to define the entire image [6].

### 4.2. Mask-Image-Projection Stereolithography (MIP-SLA)

This process, similar to stereolithography and represented by Figure 3c, is based on the localized polymerization of liquid resin through laser light exposure. Different from the projection stereolithography, a green part is preliminarily fabricated and used as a light mask to define complex profiles. In [99], for instance, the authors used the MIP-SLA process to build BaTiO_3_-based piezoelectric composite ceramics with a honeycomb structure design with a wall thickness of 450 μm.

### 4.3. Continuous Liquid Interphase Printing (CLIP)

The *continuous liquid interphase printing (CLIP),* also known as *continuous liquid interface production,* is similar to the projection stereolithography (PSLA). Figure 3d shows that the liquid resin is contained in a pool with the bottom side transparent to ultraviolet light. The light source is positioned below the transparent window; the printed object rises during the polymerization allowing other resin to flow below the exposed layer. An optically transparent membrane, permeable to oxygen, is situated below the resin [100]. The photo-polymerization is quenched by the oxygen and the membrane creates a persistent liquid interface that prevents the adhesion of the part with the pool [101].

### 4.4. Computed Axial Lithography (CAL)

In the *computed axial lithography (CAL)* there are multiple light sources, oriented along many different directions. The photoresist is contained in a cylindrical tank with transparent walls and it is exposed by 2D images along different orientations. The composition of all the exposures provides the 3D target object, according to previous calculations on the light patterns. A rotation stage can be used to obtain the same exposure condition, as represented in Figure 3e. The unexposed or underexposed resin is removed after the process. The typical features dimension is in the range 100 μm–1000 μm [102,103].

### 4.5. Digital Light Processing (DLP)

Many 3D printers are able to convert the digital object file into sliced geometry and sequential building. The *digital light processing stereolithography (DLP-SLA)* is used for building direct polymeric devices [104,105] or to support the fabrication steps of a more complicated process with different materials (Figure 3f). In [106] the DLP-SLA process is used with zirconia to build the UV-curable supports and to shape the green part, which is then sintered. The minimum feature size achieved is about 50 μm [105,106].

### 4.6. Two-Photon Polymerization (TPP) or Direct Laser Writing (DLW)

This 3D building process is based on the two-photon absorption proposed by Maruo in 1997 [107] and allows fabricating parts with extremely small dimensions, in the order of 1/100 the laser wavelength (with reference to ~810 nm wavelength of the femtosecond lasers generally used) [108,109]. In the TPP process, a laser beam transparent to the photosensitive polymer is used to generate a pulse light with high peak power (Figure 3g). When the laser is focused on a specific point, a threshold photon flux density is achieved in the middle of the focal spot [6]. The non-linear process of two-photon absorption is induced by the high-intensity light source [3]. The polymerization area (called “voxel”) is then significantly smaller than the laser wavelength [110,111,112]. The TPP allows us to control the polymerization point along the 3-dimensions with resolution below 100 nm and, for this reason, the process is also known as *direct laser writing (DLW)* [107]. The effective resolution of the process is related to many factors: laser power and wavelength, photoresist properties, oxygen diffusion, etc. [113]. The optical resolution of 5.6 nm with visible light was documented [114] and suspended lines with 9 nm feature size and interlinear resolution of 52 nm were achieved [115]. An axial resolution of 40 nm was also obtained [109].

The TPP process has recently attracted increasing attention for MEMS and NEMS (nano electro-mechanical systems) applications [116,117]; in addition, the same interaction principle between the femtolaser and the photosensitive material is applicable to other microfabrication processes as *laser ablation* [118,119], *laser melting* [120,121], and *photoreduction* [122,123].

## 5. Extrusion-Based Processes

### 5.1. Fused Deposition Modelling (FDM)

**Figure 4:**
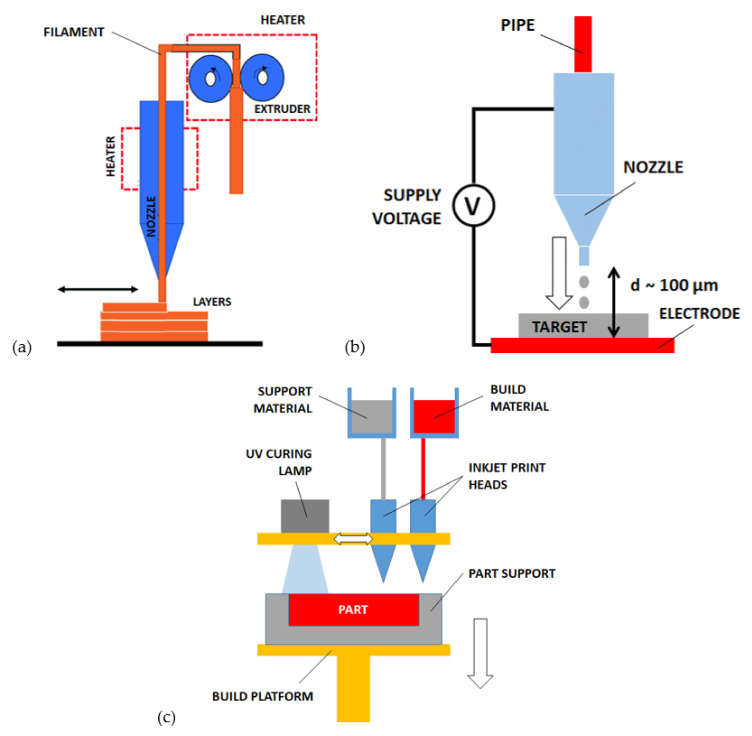
Extrusion-based processes for AM of microstructures: (**a**) fused deposition modelling (FDM), (**b**) electro-hydrodynamic ink jet printing (EHD-IJP), (**c**) multi jet modelling (MJM).

The *fused deposition modeling (FDM)*, also referenced as *fused filament fabrication (FFF)*, is a 3D printing method based on the material extrusion through a nozzle, as introduced by S. Scott Crump in 1988 [124]. An electric resistance heats and fuses the solid filament that is immediately extruded along the desired pattern to compose each layer (Figure 4a). Compatible materials are thermoplastic and thermosetting polymers, including acrylonitrile butadiene styrene (ABS), polylactic acid (PLA), and polyethylene terephthalate (PET).

In the MEMS fabrication, the limitations associated with the FDM process are the nozzle dimension and the viscosity of the materials used. The minimum size of structures is in order of 200 μm with PDMS and 400 μm with PLA and ABS.

### 5.2. Ink Jet 3D Printing (IJP)

The AM methods based on extruded materials without thermal heating are identified with the definition of *direct ink writing (DIW)* [125]. The main advantages of these methods are low cost, a large variety of materials, large printing area, and high throughput [126,127].

The *ink jet 3D printing (IJP)* is based on the deposition of small ink droplets on the substrate. Different to traditional 2D printing, the print head (or, alternatively, the substrate) is controlled also in the vertical direction. This allows building objects by superimposing successive layers [128]. The material is extruded through a nozzle and deposited along a linear pattern (continuous ink jet printing) or point-to-point (drop-on-demand ink jet printing) [129]. The most frequent method for AM is the second one [130].

The materials suitable for IJP have low viscosity and they are generally polymers, waxes, bio-inks, and hydrogels [131]. The building of 3D ink jet printed microstructures was demonstrated [132]. More specifically for microscale printing, other available materials are colloid solutions of nanoparticles, metals, composites, glasses, and biological tissues. The material is usually named “ink” in relation to this particular process [6]. The ink jet printing method allows fabricating metal electronic elements on plastic substrates thanks to the low temperatures involved and by using solder materials. Metals with low melting temperatures have been used in the jetting process to build 3D microstructures [133,134].

The low viscosity allows achieving features size of about 10 μm [135] or lower [136]. The features size also depends on the droplet’s size and nozzle diameter. The size of droplets is determined by the ejection system (or activator), normally based on piezoelectric or thermal principles. The ejection control signal (pulse shape and length) determines the formation of droplets and their size, velocity, and repeatability [137]. Other factors influencing droplets properties and objects resolution are ink surface tension, viscosity and inertia, and ink-substrate affinity. After the deposition, ink drying or annealing are provided. Extensive simulations and experiments on droplets formation with various materials are available [138].

The evolution of IJP, called *electro-hydrodynamic ink jet printing (EHD-IJP)*, reported in Figure 4b, provides the reduction of droplets size by means of an additional electric field between the printhead nozzle and the substrate [139,140]. The electric field produces a sequence of small ink volumes leaving the droplet and migrating towards the substrate. The dimension of these new droplets is much smaller than the nozzle diameter, around 100 nm. Then, the EHD-IJP is suitable for printing high-resolution micro and nano-structures (e.g., 50 nm gold nanocolumns [141] and 10 μm wax-polymer capillaries [142]) and supports the combination of multiple materials [141,143].

### 5.3. Multi Jet Modelling (MJM)

The *multi jet modeling (MJM)* is based on the parallel and simultaneous ink jet deposition of multiple materials including sacrificial support material (gray) and photocurable materials (colored). This method was introduced by Yamane et al. [144] in the 1990s and it is also referenced as “Polyjet” 3D printing. The deposition of photopolymer droplets and sacrificial material is accompanied by continuous ultraviolet (UV) photocuring, as represented in Figure 4c. The sacrificial material is generally a water-soluble gel or meltable wax, which is removed with post-processing steps [145]. Recent developments report polymer jetting processes (where aerosol jet printers are used) assisted by UV LEDs (light-emitting diodes) through focal lenses. The LEDs light is used to provide instant curing of the polymer and the lenses to focus the light beam more effectively. Polymer pillars with 20 μm diameter were built with this technique by achieving 16° light beam angle and 100 ms/pillar velocity including the curing [146].

## 6. Other Processes

### 6.1. Electron/Ion Beam Induced Deposition (E/IBID)

**Figure 5:**
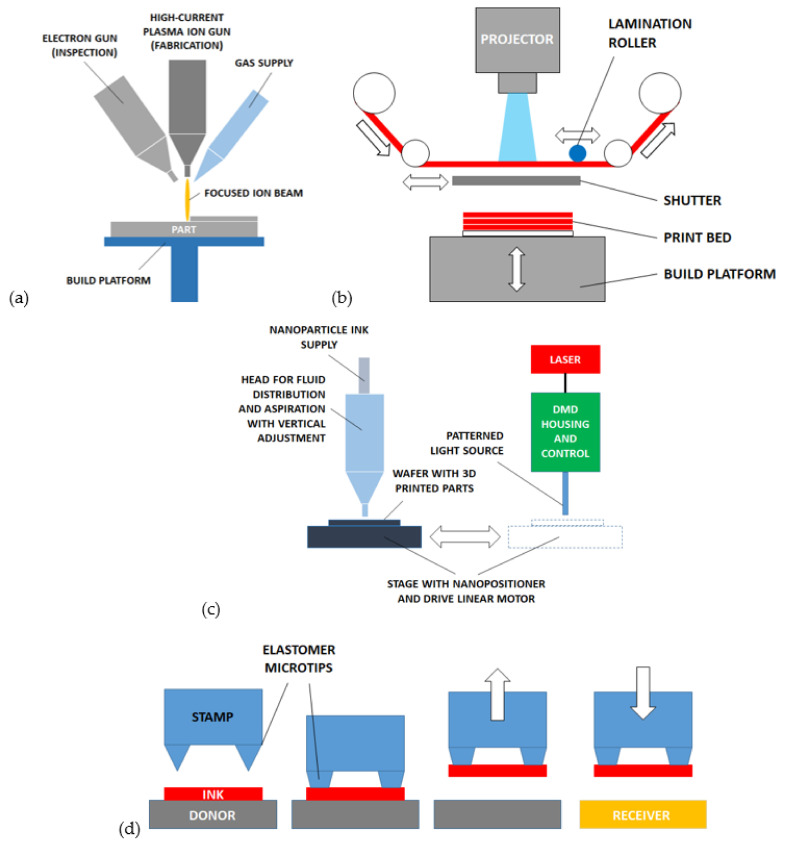
Other processes for AM of microstructures: (**a**) electron/ion beam induced deposition (E/IBID), (**b**) laminated resin printing (LRP), (**c**) ink jet selective laser sintering (IJ-SLS), (**d**) transfer printing.

The (focused) *electron beam induced deposition (EBID)* starts from a low-pressure environment with gaseous material, which is deposited on a substrate by the power source (Figure 5a). A similar alternative version uses an ion-beam source (*ion beam induced deposition-IBID*). The ideal resolution of the process is very high and related to the molecular size of the deposited material. However, many geometrical and directional constraints are present. A large variety of materials are compatible including polymers and metals [147]. The E/IBID are characterized by very low printing speed (below 1 voxel/s) [148].

An improved variant of this process, with a high impact on the MEMS scale, also called *focused ion beam (FIB)*, includes the gas supply through a nozzle close to the focused ion beam source [149]. The same ion beam is used for etching (subtractive operation) and depositing (additive operation) the material. Both insulators and metals can be processed, and high-resolution patterning is possible (below 10 μm).

### 6.2. Casting with Sacrificial Mold

The combination of different AM processes can potentially provide improved performances. The most relevant case is the preliminary fabrication of molds through various methods (SLA, SLA, SLM, etc.) followed by a casting process. In the microscale, the separation of the part from the mold is challenging and it is preferable to use sacrificial molds that are etched or dissolved after the casting. This method provides good results in particular with soft material components with complex shapes (micropillars, membranes, lattices, channels, etc.) In [12], the authors used a metal sacrificial mold with thin walls fabricated by AM to cast and cure the soft polymer (PDMS in this case). The mold is then dissolved by acids without affecting the polymer object. The challenges for MEMS fabrication are associated with the final dimensional tolerances and with parts accuracy. The tolerance is affected by the material shrinkage during the casting process, although the small volumes involved cause in general proportionally low deviations. Instead, the final accuracy of the microstructure derives from the contributions of the mold dimensional tolerances and the mold release. The sum of accuracy errors may lead to the MEMS scale of large deviations from the nominal dimensions without precise control of all the intermediate steps.

### 6.3. Laminated Resin Printing (LRP)

The LRP process is suitable for MEMS fabrication and consists in stacking many layers of dry resin previously patterned by stereolithography. As reported in Figure 5b, each layer is made by a negative photoresist that is exposed to a light source and shaped. A rapid automated process provides the sequential stacking of the resin blocks giving the growth of the 3D microstructure. The stacked layers may also work as supports for successive suspended layers forming membranes or overhangs. With reference to Figure 5b, the projector exposes the active layer while the shutter protects the building part below. The shutter is then moved and the vertical stage translates upwards so that the part and the new layer are put in contact. The heated roller provides the pressure needed for lamination, then the stage returns in a down position and the shutter returns to protection mode.

Complex shapes with low cost and fast cycle time are feasible with LRP, and post-processes as metallization, etching, or microfabrication steps are applicable. Small machines composed of a DLP projector and lamination system (shutter and movable stage) support the process. Typical resolutions achievable are in the order of 10 μm with 1920 × 1080 resolution projectors [150].

### 6.4. Ink Jet Selective Laser Sintering (IJ-SLS)

This process is the evolution of selective laser sintering (SLS) and ink jet printing (IJP). The special setup of this process allows achieving probably the highest resolutions in metal 3D printed microstructures (in the order of 5 μm) and the fastest velocities (about 60 mm^3^/h). The process, described in [54] and represented in Figure 5c, provides the preliminary substrate coating with ink including metal nanoparticles inside. The layer of ink is then dried and exposed to a light source by means of nano-positioners for high precision alignment. The laser light beam used for the exposition is preliminary patterned by DMDs. The exposure causes metal particles to sinter and form a layer formation. The process is repeated to build multiple stacked layers.

### 6.5. Transfer Printing

The transfer printing process provides the translation of solid material blocks from a donor substrate (where the material is preliminary grown or deposited) to a receiving substrate (Figure 5d) [151,152,153]. The solid materials transferred are also called “solid inks”. The transfer is performed through an elastomeric stamp with sharp tips provided with shape memory. The flexible stamp is contacted and preloaded upon the solid material to induce local adhesion with the sharp tips. The stamp-ink assembly is then moved to the target position and heated. The thermal heating restores the original shape of stamp tips and the solid ink is released. The process was initially applied to single-crystal silicon inks (namely “*micro-masonry*”), then extended to silicon dioxide, gold, and epoxy polymer (SU8). The process is also known as *micro-LEGO* and supports many geometrical variants and configurations for the creation of MEMS assemblies (rotors [154], comb-drives [155], micromirrors [156], resonators [157], and cantilevers [158]).

## 7. Discussions

The AM of microstructures, similarly to the macroscale field, allows the fabrication of complicated shapes that are impossible to obtain with other processes. The AM methods also permit to use a large variety of materials and materials combinations [159,160,161,162,163]. Some of the processes described have been developed by starting from the macroscale to increase their performances and resolutions, making them suitable for building microstructures and MEMS. The comparison between AM at micro and macro scales shows significant differences among the two fields. The fabrication of structures at the micrometer range requires many steps including traditional micromachining methods and AM. Then, the integration and compatibility of these production phases is very significant (e.g., the deposition of conductive electrodes on structural parts) and causes strong constraints to the spread of AM that are not present in the macroscale. The standardization and stabilization of many processes are almost mature at the macroscale, while large variability of results still exists in the microscale, as the literature demonstrates. The constant advantage of AM is the possibility to build structures with complex shapes not allowed by conventional methods. The design digitalization provides high potential in managing complex MEMS projects with high flexibility. This characteristic of AM also leads to the advantage related to small volume production and parts parallelization, where small variations among samples are possible.

Reasonably, the most promising AM processes for future evolution are those ones able to extend their resolution up to the nanometer range and suitable for the simultaneous or parallel building of 3D structures. Among these methods there is TPP (two-photon polymerization). The ink jet process is also promising, in combination with other methods as IJ-SLS (ink jet selective laser sintering) or combined with subtractive processes to provide hybrid fabrication.

The next perspectives of AM in the micrometer field are related to the following expected advancements of the technologies [6]. Firstly, future processes will require higher resolutions and will be targeted to the molecular scale. The quality of parts, referring to dimensional accuracy, roughness, and process repeatability, need to be improved. New materials, specifically designed and developed for AM, are needed to improve the functional properties of structures. The AM processes themselves require enhanced efficiency, higher throughputs (more parts per unit time), and parallelization. The AM process will be integrated into larger high-performance processes to cover the entire MEMS fabrication. Finally, dedicated tests and in-line controls are required for process management and quality monitoring. On the contrary, the traditional micromachining processes will experience advancements on the typologies of compatible materials, on the processes throughput and cost reduction of high volumes productions.

**Figure 6:**
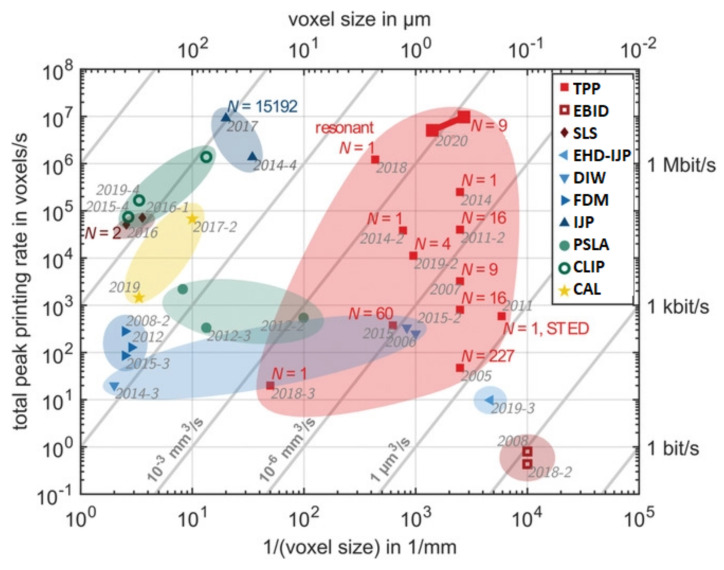
Performances comparison among AM processes in terms of printing velocity (left vertical axis) and printing resolution (lower horizontal axis) [148].

With reference to the mentioned process efficiency and throughput, associated with parts details resolution, Figure 6, adapted from the original version published in [148], reports the comparison of performances among the AM processes. Two figures of merit are reported: “faster” and “finer”. The left vertical axis reports the printing speed in terms of the number of voxels per second (“faster”), and the lower horizontal axis reports the inverse voxel size (“finer”). Additionally, the upper horizontal axis is the voxel size (10 nm–1 mm) scale, and the right vertical axis is the bit rate (in bits/s) at which the digital information is converted into hardware.

More in detail, the definition of each process in terms of minimum feature size documented and available materials are listed in Table 1. The next Table 2 reports the most relevant ceramic materials used in MEMS processed with AM.

## 8. Conclusions

The most important AM processes suitable for building microstructures and MEMS are identified and described with the goal being to organize the updated information associated with the state of the art of this field.

The AM at the micrometer scale is producing some preliminary relevant examples demonstrating the processes performances and their potential. These experiences are diffused in academia and industry but globally they are still considered as pioneering exploration related to an embryonal field waiting for long-term evolution. Today, the fabrication speed and production parallelization issues are limiting the AM technologies to stand-alone microsystems or case studies. Furthermore, the limitations related to the simultaneous fabrication of electrically conductive parts and insulating parts are reducing the applicability of the AM processes not supported by other traditional steps. The next-future progress of additive technologies must necessarily overcome these issues to expand their applicability. Additionally, it is not reasonable to push the AM to replace extensively the conventional micromachining processes, which have undoubtedly superior performances. Instead, the future MEMS designers will probably exploit the specific performances of novel methods when they are unique and without alternatives. For this purpose, the combination of consolidated and innovative skills and competencies will be required for the next generation of MEMS designers. The knowledge of AM processes details and of electro-mechanical design opportunities provided by the freeform design will be crucial. This will lead to the development of design skills dedicated to the AM of microstructures, not limited to alternative ways to provide the same output of conventional processes but able to sustain the real technological evolution of MEMS.

The processes described in this paper are always associated with the digitalization of the production, which is probably the next improvement expected in manufacturing at the micrometer range. In fact, 3D printing is associated with geometries preliminary optimized through digital operations and then converted to physical objects. This also preludes to the transition from centralized computation to individual distributed manufacturing supported by personal computers and local efforts. Furthermore, another typology of digitalization, called the Internet of Things (IoT) will interest most of the manufacturing areas in the future and it will be powered by MEMS, preliminarily based on the AM process. MEMS-based devices will support the monitoring and management of tools, systems, physical parameters, wearable devices, machines, and buildings.

## Figures and Tables

**Table 1:** Performances and suitable materials of AM processes for microstructures fabrication.

Process	Min. Feature (μm)	Materials	References
Powder-based processes			
Multijet fusion (MJF)	250–500	Polymers (PA11, PA12, TPU)	[35,46,47,48,164]
Selective laser sintering (SLS)	40–100	Polymers, metals, ceramics, hydroxyapatite, glasses	[13,49,50,53,95,165,166,167,168]
Selective laser melting (SLM)	40–200	Metals	[73,167,168,169,170,171]
Electron beam melting (EBM)	100–200	Metals	[73,168,170]
Powder bed binder jetting (PBBJ)	100 (metals, polymers) 22–500 (ceramics)	Polymers, metals, ceramics, composites, sand	Polymers, metals [13,79,95,165,166,167] Ceramics, composites [13,15,79,95,166,167,172,173]
Powder directed energy deposition (PDED)	500–3000	Metals	[73,168,170]
Other laser-based processes			
Micro-stereolithography (μ-SLA)	30–70	Photosensitive polymers, Formlabs clear resin	[174,175]
Mask-image-projection stereolithography (MIP-SLA)	450	Photosensitive polymers	[99]
Continuous liquid interphase printing (CLIP)	100	Photosensitive polymers	[100,101]
Computed axial lithography (CAL)	100–1000	Photosensitive polymers	[102,103]
Digital light processing (DLP)	50	Polymers, ceramics	[104,105,106]
Two-photon polymerization (TPP) or Direct laser writing (DLW)	0.085–1.5 (photoresist) 25 (Poly-diacrylate)	Photoresist, Poly-diacrylate	[108,109,176,177,178]
Extrusion-based processes			
Fused deposition modeling (FDM)	200 (PDMS) 400–500 (PLA, ABS)	Polymers	[179,180,181,182,183]
Ink jet 3D printing (IJP)	10–200	Polymers, metals	Polymers [131,184] Metals [135,136]
Multi jet modelling (MJM)	20–50	Photosensitive polymers	[144,145,146]
Other processes			
Electron/ion beam induced deposition (E/IBID)	10	Polymers, metals	[147,148,149]
Casting with sacrificial mold	150–500	Polymers	[12,104]
Laminated resin printing (LRP)	10	Polymers, metals	[150]
Ink jet selective laser sintering (IJ-SLS)	5	Metals	[54]
Transfer printing	100	Single crystal silicon, silicon dioxide, gold, SU8	[151,152,153]

**Table 2:** Fundamental ceramic materials suitable for AM processes.

Material	Process	Resolution	Reference
Alumina	Micro-stereolithography (μ-SLA)	150 nm	[185,186,187,188]
	Digital light processing (DLP)	100 μm	[189,190]
	Selective laser sintering (SLS)	50 μm	[191,192,193]
	Ink jet 3D printing (IJP)	100 μm	[194]
SiC	Casting with sacrificial mold	76 μm	[195]
	Selective laser sintering (SLS)	50 μm	[191,192,193,196]
	Sheet lamination	-	[197]
Hydroxyapatite	Fused deposition modeling (FDM)	200 μm	[198]
Zirconia	Micro-stereolithography (μ-SLA)	150 nm	[185,186,187,188]
	Ink jet 3D printing (IJP)	100 μm	[194]
Polymer derived ceramics	Micro-stereolithography (μ-SLA)	150 nm	[185,186,187,188,199]
	Digital light processing (DLP)	100 μm	[189]
	Continuous liquid interphase printing (CLIP)	64 μm	[200]

## Nomenclature

ABS	acrylonitrile butadiene styrene
AM	additive manufacturing
CAD	computer aided design
CAL	computed axial lithography
CLIP	continuous liquid interphase printing
DIW	direct ink writing
DLD	direct laser deposition
DLF	direct light fabrication
DLMD	direct laser metal deposition
DLP	digital light processing
DLW	direct laser writing
DMD	digital micro-mirror device
DMLM	direct metal laser melting
DMLS	direct metal laser sintering
DSLS	direct selective laser sintering
EBID	electron beam induced deposition
EBM	electron beam melting
EHD-IJP	electrohydrodynamic ink jet printing
FDM	fused deposition modelling
FIB	focused ion beam
FFF	fused filament fabrication
IBID	ion beam induced deposition
IJP	ink jet 3D printing
IJ-SLS	ink jet selective laser sintering
LDW	laser deposition welding
LED	light-emitting diode
LENS	laser engineered net shaping
LPBF	laser powder bed fusion
LMD	laser metal deposition
LRP	laminated resin printing
MEMS	micro electro-mechanical systems
MIP-SLA	mask-image-projection stereolithography
MJF	multi jet fusion
MJM	multi jet modelling
NEMS	nano electro-mechanical systems
PA	polyamide
PBBJ	powder bed binder jetting
PBF	powder bed fusion
PBF-LB/M	laser-based powder bed fusion of metals
PBF-LB/P	laser-based powder bed fusion of polymers
PDED	powder directed energy deposition
PDMS	polydimethylsiloxane
PET	polyethylene terephthalate
PFW	powder fusion welding
PLA	polylactic acid
PSLA	projection stereolithography
PZT	lead zirconate titanate
SLA	(micro) stereolithography
SLM	selective laser melting
SLS	selective laser sintering
SLSLA	scanning laser stereolithography
TPP	two-photon polymerization
UV	ultraviolet
VED	volume energy density
